# ATR-FTIR Spectroscopy for the Assessment of Biochemical Changes in Skin Due to Cutaneous Squamous Cell Carcinoma

**DOI:** 10.3390/ijms16046621

**Published:** 2015-03-24

**Authors:** Cássio A. Lima, Viviane P. Goulart, Luciana Côrrea, Thiago M. Pereira, Denise M. Zezell

**Affiliations:** 1 Center for Lasers and Applications, Instituto de Pesquisas Energéticas e Nucleares, IPEN-CNEN/SP, São Paulo SP 05508-000, Brazil; E-Mails: cassiolima@usp.br (C.A.L.); vi.pgoulart@gmail.com (V.P.G.); thiagomartinipereira@gmail.com (T.M.P.); 2 School of Dentistry, Universidade de São Paulo, São Paulo SP 05508-000, Brazil; E-Mail: lcorrea@usp.br; 3 Instituto de Ciência e Tecnologia (ICT), Universidade Federal de São Paulo, São José dos Campos SP 12.231-280, Brazil

**Keywords:** squamous cell carcinoma, optical diagnosis, laser spectroscopy, ATR-FTIR spectroscopy, hierarchical cluster analysis

## Abstract

Nonmelanoma skin cancers represent 95% of cutaneous neoplasms. Among them, squamous cell carcinoma (SCC) is the more aggressive form and shows a pattern of possible metastatic profile. In this work, we used Fourier transform infrared spectroscopy (FTIR) spectroscopy to assess the biochemical changes in normal skin caused by squamous cell carcinoma induced by multi-stage chemical carcinogenesis in mice. Changes in the absorption intensities and shifts were observed in the vibrational modes associated to proteins, indicating changes in secondary conformation in the neoplastic tissue. Hierarchical cluster analysis was performed to evaluate the potential of the technique to differentiate the spectra of neoplastic and normal skin tissue, so that the accuracy obtained for this classification was 86.4%. In this sense, attenuated total reflection (ATR)-FTIR spectroscopy provides a useful tool to complement histopathological analysis in the clinical routine for the diagnosis of cutaneous squamous cell carcinoma.

## 1. Introduction

According to the World Health Organization (WHO), by 203,027 million new incident cases of cancer could be expected, 17 million cancer deaths annually and 75 million persons alive with cancer within five years of diagnosis [1]. The most common form of malignancy in humans is nonmelanoma skin cancer (NMSC) and represents nearly 95% of all cutaneous neoplasms [2]. In Brazil, according to the 2014 report of the National Cancer Institute, 30% of cases predicted are represented by NMSCs [3].

Most NMSCs are related to ultraviolet (UV) [2,4,5] light exposure and due to the heterogeneity of the skin, they may have different tumor lines. The most common types are basal cell carcinoma (BCC) and squamous cell carcinoma (SCC). Approximately 75% of diagnostics cases of NMSCs are related to BCC and 20% for SCC. Although more common, BCC is less aggressive and rarely presents metastasis. On the other hand, SCC is aggressive and shows a pattern of destructive growth with a metastatic profile.

Considering that early diagnosis has fundamental importance for a treatment with favorable results, the search for techniques that can provide supplementary information for physicians in detection of neoplasms in early stages have been subject of several scientific works [6,7,8]. In this sense, Fourier transform infrared spectroscopy (FTIR) can provide biochemical information complimentary to the morphological histopathology, using the same biopsy material. Biological molecular bonds with an electric dipole moment that can change by atomic displacement due to natural vibrations are infrared active and therefore are quantitatively measured by infrared spectroscopy [7].

FTIR spectra can be acquired mainly in three different experimental configurations: transmission, reflection-absorption (transflection) or attenuated total reflection (ATR) [9]. The first one operates by transmitting IR radiation through sample-substrate, whereas in transflection the radiation interacts with sample and is back reflected off by the substrate [9]. Finally, on the ATR mode the sample is placed on a crystal with refractive index higher than sample, inducing total internal reflection of incident radiation, which is attenuated and penetrates into the sample as an evanescent wave [9,10].

Previous studies have discussed spectra alterations associated with transmission and transflection sampling mode. In transflection, the low-emissivity slides commonly used as reflector substrates contribute to formation of a standing wave perpendicular to sample surface that leads to spectral changes not related to the biochemistry of sample [11,12,13]. In transmission mode, spectra collected are subject to a variety of physical effects occurring in the same time with absorption, and requires corrections for phenomena, such as light scattering [14], refraction and dispersion [15], and other optical effects specifically relative to measuring thin films, which may also induce spectral distortions [16]. However, these unwanted spectral contributions are not presented in the ATR sampling mode [9], which provides to be a simple and powerful tool for analyze liquids and thin films samples with no preparation method [10]. ATR-FTIR spectroscopy provides a single spectrum, which represents the average signal from the sample area that light passed through [10]. Added to this, ATR sampling mode presents high SNR (signal-to-noise ratio) compared to those obtained by transflection and transmission configurations [7]. Measurements in ATR-FTIR spectroscopy may be performed as macro ATR-FTIR or fiber-ATR, which can be used in the future, directly on a patient’s skin [17,18,19].

## 2. Results

### 2.1. Histopathological Analysis

**Figure 1:**
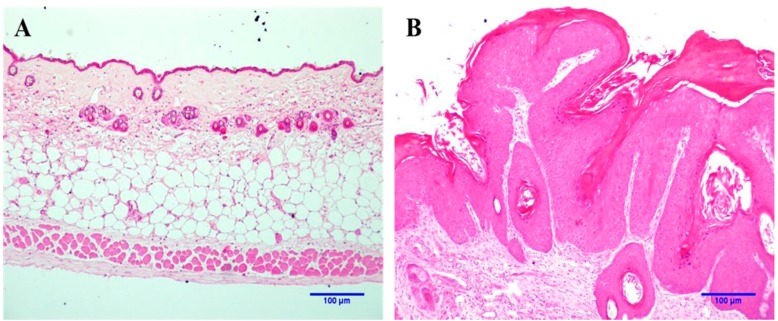
Light microscopy of representative histological sections hematoxylin–eosin (H&E) stained; (**A**) Healthy skin and (**B**) Neoplastic lesion.

The anatomopathological characteristics presented in a biological tissue is the gold standard for diagnosis of NMSCs. Figure 1 shows the profile of normal and neoplastic skin lesions induced by multi-stage chemical carcinogenesis in mice obtained by histopathological analysis.

The anatomopathological characteristics for both tissues are clearly different. The epidermis of normal skin is consisted of three or four cell layers covered by a thin stratum corneum (Figure 1A), whereas neoplastic lesions show an intense proliferation of keratinocytes in an exophytic profile, covered by a thick stratum corneum (Figure 1B). Added to this, moderate dysplasia characterized by an intense nuclear hyperchromatism and cell pleomorphism was present in the epithelial basal layer of neoplastic lesions. The exophytic pattern of the epithelium associated with epithelial dysplasia was compatible with DMBA (7,12-dimethyl-benzanthracene)/TPA (12-*O*-tetradecanoyl-phorbol-13-acetate)-induced neoplastic lesion in the skin.

### 2.2. ATR-FTIR Spectroscopy

**Figure 2:**
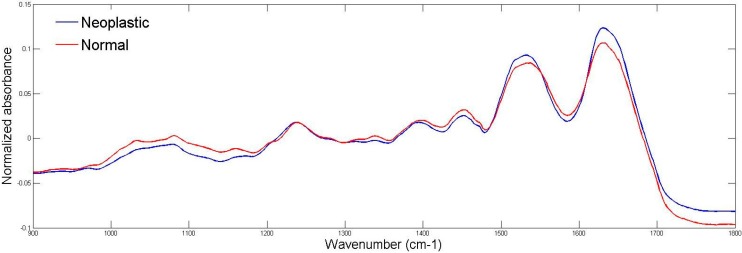
Fingerprint region (900–1800 cm^−1^) of normal (red line) and neoplastic lesions (blue line).

ATR-FTIR spectroscopy was used to compare neoplastic lesions with normal skin tissues. Figure 2 shows the spectra from 900–1800 cm^−1^ (fingerprint region), which provides information of vibrational modes associated with important cell content.

**Figure 3:**
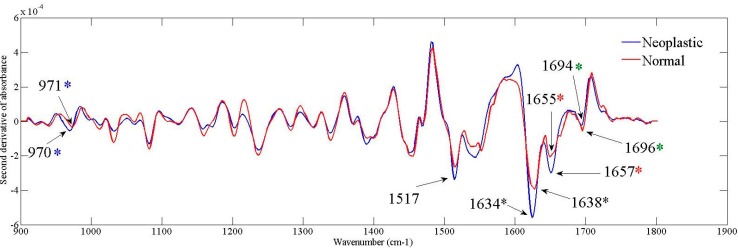
Second derivative of averaged spectra obtained for the neoplastic and normal tissues. The asterisks depict the shifts presented for the bands: Blue asterisk for 970 cm^−1^; Black asterisk to 1634 cm^−1^; Red asterisk for 1657 cm^−1^; Green asterisk to 1694 cm^−1^.

Due to the overlapping of sub-bands in the row spectra, we calculated the second derivative of absorbance to compare the averaged spectra, as shown in Figure 3.

Both groups showed similar vibrational modes, in which some differences in the absorption intensities were observed, as well as shifts in the peak position.

Table 1 shows the band position of vibrational modes statistically different identified in the fingerprint region. The mean band intensity and its respective standard deviation are presented in the third and fourth columns, respectively, and the last column shows the p value, thus indicating the differences between bands.

Band peaking at 971 cm^−1^ is related to symmetrical stretching mode of dianionic phosphate monoesters of phosphorylated proteins and nucleic acids [20,21,22]. Increased in its intensity was observed in neoplastic tissue compared to normal skin, as well as a slight shift for 970 cm^−1^ as depicted by blue asterisk in Figure 3.

**Table 1:** Observed wavenumber values and their statistical comparison between assignments normal and neoplastic tissue, and the *p* values (*t*-student test).

ATR-FTIR Band Positions (cm^−1^)		Band Intensity		*p* Value
Normal	Neoplastic	Normal	Neoplastic	
971	970	−3.919 × 10^−5^ ± 3.853 × 10^−6^	−5.8 × 10^−5^ ± 3.536 × 10^−6^	0.0007
1517	1517	−2.68 × 10^−4^ ± 5.0 × 10^−6^	−3.49 × 10^−4^ ± 8.726 × 10^−6^	0.0001
1638	1634	−4.1 × 10^−4^ ± 9.918 × 10^−6^	−5.64 × 10^−4^ ± 1.393 × 10^−5^	0.0001
1655	1657	−2.14 × 10^−4^ ± 5.333 × 10^−6^	−3.07 × 10^−4^ ± 1.430 × 10^−5^	0.0001
1694	1696	−5.641 × 10^−5^ ± 2.538 × 10^−6^	−2.407 × 10^−5^ ± 2.270 × 10^−6^	0.0001

Spectral range from 1500–1700 cm^−1^ provide information peptide bonding in proteins and its secondary structure. Band peaking at 1517 cm^−1^ arise in the neoplastic tissue and it is reported to amide II vibration [20], which results from the combination out-of-phase of the N–H in plane bend and the C–N stretching vibration with smaller contributions from the C–O in plane bend and the C–C and N–C stretching vibrations [23]. Bands located in 1638 cm^−1^ [24], 1655 cm^−1^ [20,23] and 1694 cm^−1^ [20,23] represent the amide I vibrations, characterized mainly from the C=O stretching with minor contributions from the out-of-phase C–N stretching, the C–C–N deformation and the N–H in-plane bend [20,23]. Bands at 1638 and 1694 cm^−1^ are associated with the β-sheet secondary structure of proteins, more specifically to parallel and anti-parallel configuration. As shown in Figure 3, the vibrational mode associated to parallel β-sheet increased its intensity and shifted to a lower wavenumber (black asterisk) in the neoplastic tissue. On the other hand, the anti-parallel β-sheet shifted to a higher wavenumber (green asterisk) and decreased its intensity in the tumor spectra. Finally, the band in 1655 cm^−1^ is associated to the vibrations of the α-helix structure of proteins and displays an increase in neoplastic tissue and it is shifted for a high wavenumber peak (red asterisk).

### 2.3. Cluster Analysis

**Figure 4:**
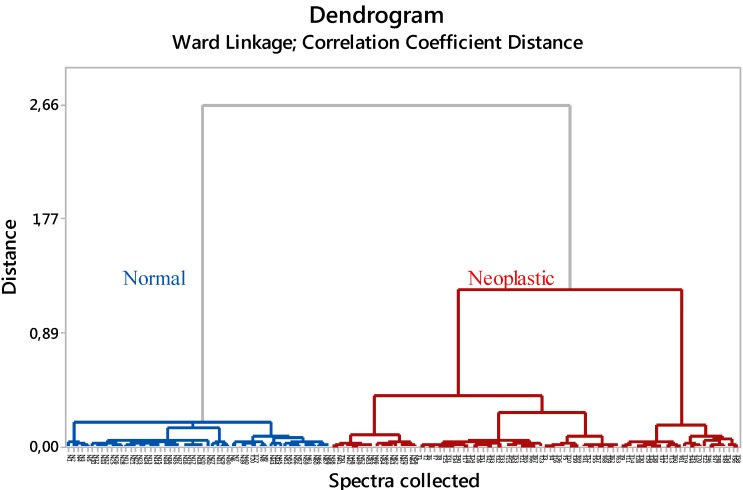
Classification of the dataset into two categories (normal in blue and neoplastic in red).

Spectra of neoplastic and normal skin were pre-processed prior to the application of hierarchical cluster analysis (HCA) that was used as an unsupervised classification technique aiming at sorting the spectra into two categories. HCA was performed to evaluate the potential of the technique to differentiate the spectra of neoplastic and normal tissue. For this, second derivative of ATR-FTIR spectra were used to minimize linear baseline effects and correct quadratic effects caused by particle scatter. Results of clustering are displayed in a tree-like diagram called dendrogram, so that spectra within a same cluster should describe spectra with similar characteristics. Results of FTIR spectra classification are shown in Figure 4.

The dendrogram shown in Figure 4 classify all data into two groups. Samples named T1–T70 correspond to the spectra obtained for the tissue extracted from animals submitted to carcinogenesis (neoplastic group), whereas those named as N1–N70 represent the spectra obtained for the normal group. The way that the data are distributed in the groups is shown on the abscissa axis and the distance of spectra within the same cluster is shown on the ordinate axis.

Considering the distribution of the data into the clusters obtained with HCA, the accuracy, sensitivity and specificity of classification was calculated as shown in Table 2.

**Table 2:** Distribution of the dataset in groups for the calculus of the accuracy of clustering classification.

	True Positive (TP)	True Negative (TN)	False Positive (FP)	False Negative (FN)	Accuracy (%)	Sensitivity (%)	Specificity (%)
Spectral data	68	53	17	2	86.4	97.1	75.7

For the calculus, we considered the true positive as the neoplastic spectra in the neoplastic group; true negative as the normal spectra in the normal group; the false positive as normal spectra in the neoplastic group; and false negative as neoplastic spectra in the normal group. The accuracy obtained for the classification of all dataset was 86.4%, sensitivity was 97.1%, and specificity 75.7%.

## 3. Discussion

As previously described, the fingerprint spectral region provides important information related to the biochemistry of analyzed tissue. However, it is noticeable that the tissue-sample analysis is much more complex than the simple explanation of the features of single component vibration. In a biological system the effect of each structure may interact with the others and result in the amplification or reduction of a specific signature.

Band absorption at 970 cm^−1^ is assigned to vibrational modes of phosphorylated proteins, and it is increased for neoplastic tissue. Phosphorylation or dephosphorylation process can affect the function of a protein in every conceivable way, increasing or suppressing activity, marking a protein for destruction, allowing it to move from one subcellular compartment to another, or enabling it to interact with or dissociate from other proteins [25,26,27]. In this sense, phosphorylation processes are an important signaling pathway involved in processes related to cancer development. These protein changes can be assessed by FTIR spectra using information provided by vibrations associated to amide region, which is highly active and sensitive in infrared.

Our findings indicated an increase in amide II band at 1517 cm^−1^ and suggest high level of protein expression, which may be explained by the intense metabolic activity of neoplastic tissue and its high demand for proteins performing signaling pathways for proliferation of cancer cell, oncogenic kinases signaling, transcriptional regulation, and other functions.

Sub-bands of amide I related to α-helix (1655 cm^−1^) and parallel β-sheet (1638 cm^−1^) structures of proteins showed a higher increase and shifted in neoplastic tissue compared to normal skin. Parallel β-sheet shifted its position 4 cm^−1^ to a lower wavenumber direction (black asterisk in Figure 3), whereas a shift of 2 cm^−1^ in the direction of a higher wavenumbers was found for α-helix structure (red asterisk in Figure 3). These results suggest changes in hydrogen bonding between peptide groups and consequently in molecular geometry of proteins, which may induce damages in protein folding and result in definitive loss of protein biological function or mutation.

## 4. Experimental Section

### 4.1. Chemical Carcinogenesis

For induction of the neoplasia, we used a well-established *in vivo* model of chemically-induced skin tumor on mice. We used 16 Swiss female mice, aged from 8 to 10 weeks, with a weight of 20 g. The animals were anesthetized with ketamine (0.35 mL/kg) and xylazine (0.20 mL/kg) during all stages of the protocol, which was approved by the ethics committee for research on animals (Comite de Etica no Uso de Animais, CEUA) of Instituto de Pesquisas Energéticas e Nucleares (IPEN) (project no. 71/10-CEUA-IPEN/SP, 21 December 2010). The mice were divided into 2 groups (Table 3).

**Table 3:** Mice groups.

Group	Treatment	Description
Group 1 (*n* = 13)	Normal	Normal skin
Group 2 (*n* = 13)	Neoplasia	Neoplastic skin

**Figure 5:**
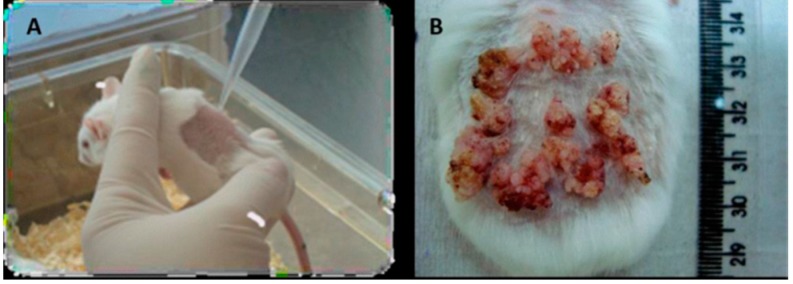
Macroscopic view of the skin lesions. (**A**) Topical application of carcinogenic substance in mouse skin; (**B**) Neoplastic lesions obtained with chemical carcinogenesis after 28 weeks.

The chemical carcinogenesis consisted of two stages. The initiation phase was a topical application on the shaved backs of 50 mg DMBA (7,12-dimethyl-benzanthracene) diluted in 100 mL of acetone. The promotion phase began one week after and consists in a bi-weekly application of 5 g of TPA (12-*O*-tetradecanoyl-phorbol-13-acetate) diluted in 200 mL of acetone, during 28 weeks. After 28 weeks, the animals obtained visible single or multiple tumor nodules with verrucous aspect (Figure 5). The control group only received topical application of acetone [28].

Tissue was extracted by biopsy and kept in formaldehyde for 24 h to the histological fixation. Once fixed, the biological tissues were diaphanized in two baths of pure xylol for 30 min and dehydrated with ethanol baths in increasing concentrations (50%, 70% and 100%). After the dehydration, the samples were mounted in wax blocks and slices of 5 µm thickness were obtained from FFPP (Formalin-fixed paraffin-processed) sections using a microtome and placed in MirrIR low-E-coated slides (Kevley Technologies, Chesterland, OH, USA) for the spectroscopic analysis.

Due to the spectral contributions of paraffin in the used wavenumber range, FFPP sections were submitted to dewaxing protocol [29]. For this purpose, FFPP sections were immersed in a series of baths consisting of two baths of xylene during 10 min and one bath of absolute ethanol during 5 min. After this, samples were kept in a desiccator for 24 h.

### 4.2. ATR-FTIR Spectroscopy

ATR-FTIR measurements, in the range 4000–400 cm^−1^, with 4 cm^−1^ of spectral resolution, were recorded using an Attenuated Total Reflectance (Smart Orbit, Thermo Scientific, Waltham, MA, USA) accessory coupled to a Fourier transform infrared spectrometer (Thermo Nicolet 6700, Waltham, MA, USA) system. The samples were pressed into the diamond crystal of ATR with a standardized pressure using a manometer. FTIR spectrometer was fitted with a deuterated triglycine sulfate (DTGS) detector (Thermo Scientific). For each spectrum, 100 scans were co-added and the spectrum obtained for each sample represents the averaged from 10 replicates measured in each sample. The Thermo Scientific system was controlled with Omnic software (Thermo Scientific).

Second derivative of spectra were smoothed with a Savitzky–Golay filter with a polynomial of second order in an eleven points window. Hierarchical cluster analysis (HCA) was used as an unsupervised classification technique in order to evaluate the similarity level between spectral data structures. The similarity of different clusters was defined by correlation coefficient distance and calculated by Ward’s method in the second derivative spectrum for each sample using software Minitab 17 (Minitab Inc., State College, PA, USA).

## 5. Conclusions

The current study shows that clustering analysis showed 86.4% accuracy in classifying spectra of neoplastic lesions from normal tissue, which presented mainly differences in the wavenumbers associated with the protein content. In this sense, ATR-FTIR spectroscopy provides a useful tool to complement histopathological analysis in the clinical routine for the diagnosis of SCC.
